# Two ophthalmic presentations of chronic myeloid leukaemia

**DOI:** 10.1002/jha2.461

**Published:** 2022-05-06

**Authors:** Miles Kiernan, Hanna Renshaw, Danny Mitry, Fion Bremner

**Affiliations:** ^1^ Oculoplastics Service Royal Free Hospital London UK; ^2^ Haematology Service Royal Free Hospital London UK; ^3^ Vitreoretinal Service Royal Free Hospital London UK; ^4^ Neuro‐Ophthalmology Service National Hospital for Neurology and Neurosurgery London UK

1

Chronic myeloid leukaemia (CML) is a myeloproliferative neoplasm that accounts for 15% of newly diagnosed cases of leukaemia in adults [1]. In the developed world, 50% of patients are asymptomatic at diagnosis with the disease picked up on routine examination and blood testing. At presentation, 90%–95% of patients have chronic phase CML during which signs and symptoms typically result from anaemia and splenomegaly [2]. Ophthalmic presentations of CML are uncommon, nonetheless clinicians should be alert to CML presenting with eye signs and the pathophysiology that underpins these findings.

The first case (Figure 1) is of a 53‐year‐old man who reported 10 days of bilateral blurred vision and palpitations on exercise. He had no significant past medical history. His visual acuities were 6/7.5 right and 6/12 left. Fundus examination revealed peripheral perivascular sheathing and florid bilateral Roth spots (Figure 1A,B). He had cystoid macular oedema and leukaemic infiltrates, confirmed on optical coherence tomography. His white blood count (WBC) was 391.4 × 10^9^/l, haemoglobin 83 g/l and platelets 200 × 10^9^/l.

**FIGURE 1:**
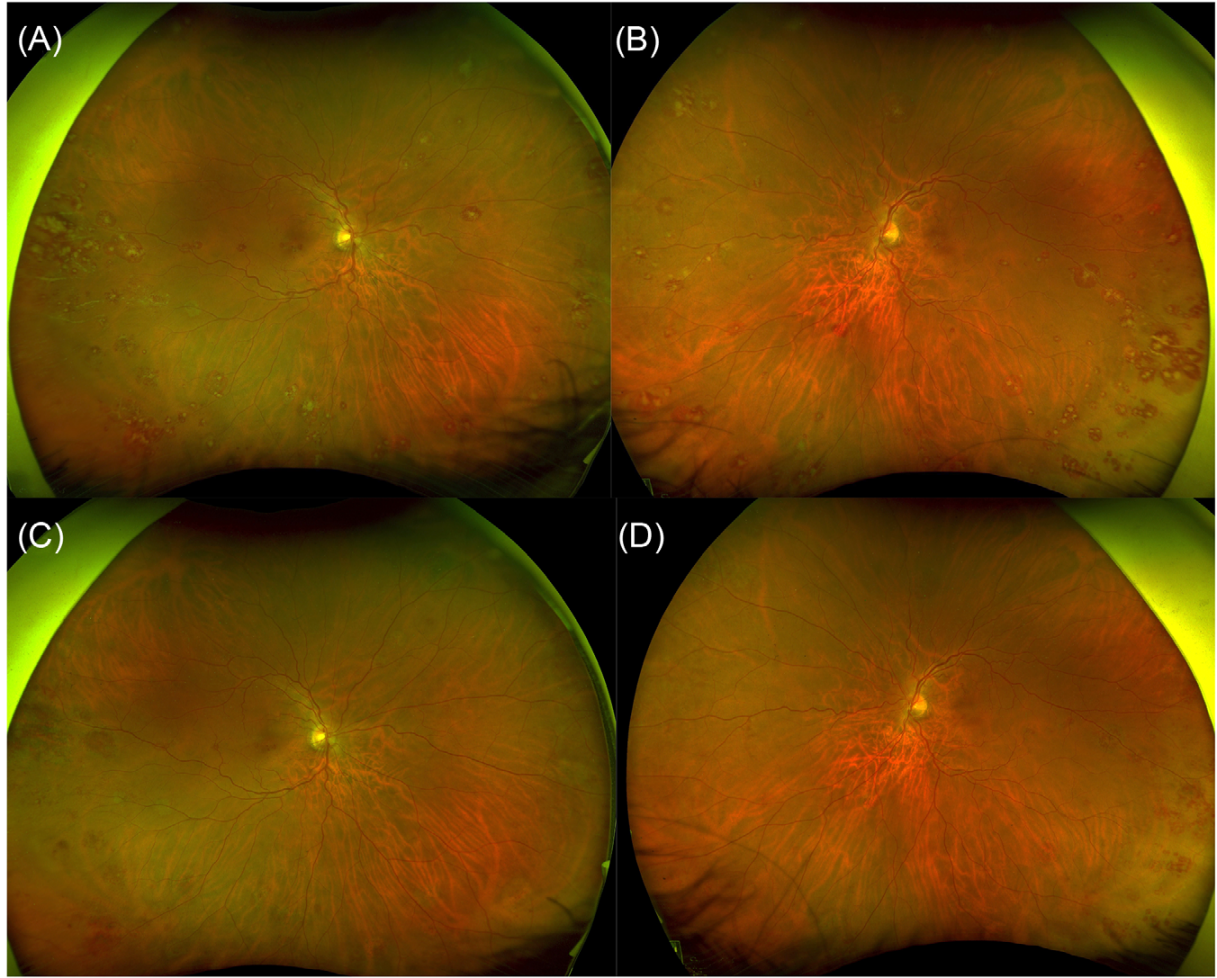
(A and B) Right and left wide‐field fundus images showing bilateral Roth spots and peripheral perivascular sheathing. (C and D) Improvement at 4 weeks following treatment

The second case (Figure 2) is of a 17‐year‐old man who described a 10‐month history of headaches and lethargy. He had no significant past medical history. He was tachycardic (heart rate 120/min), and the ophthalmic examination revealed visual acuities of 6/6 in both eyes, bilateral optic disc swelling with corresponding blind spot enlargement on visual field testing and tortuous congested retinal vessels. His WBC was 645.8 × 10^9^/l, haemoglobin 72 g/l and platelets 589 × 10^9^/l, and a CT head was reported as showing no intracranial pathology.

**FIGURE 2:**
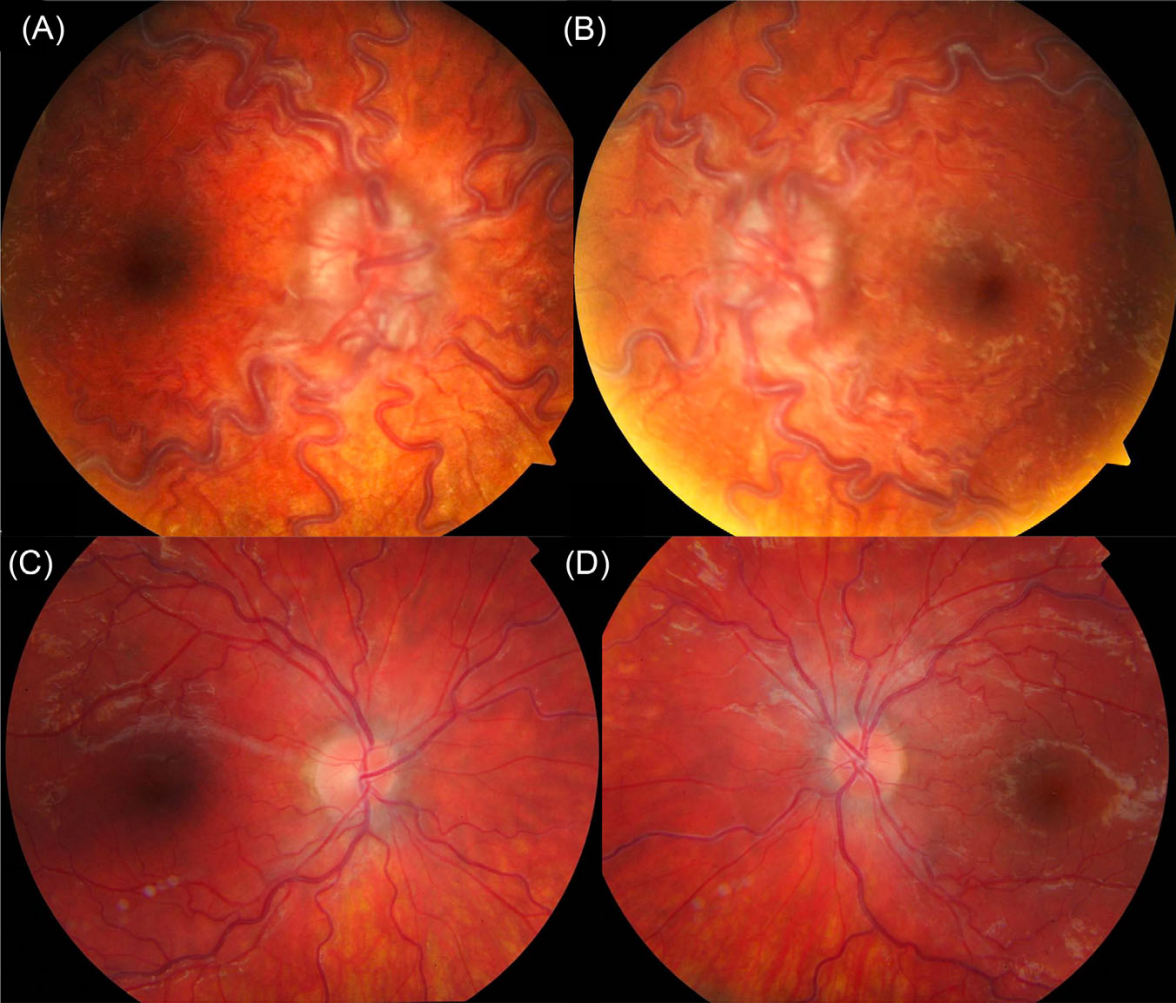
(A and B) Right and left fundus photographs showing bilateral disc swelling and vessel tortuosity. (C and D) Improvement at 6 weeks following treatment

In both cases, the peripheral blood film was consistent with CML, and cytogenetic analysis of the bone marrow aspirate detected the Philadelphia chromosome. The patients received oral hydroxycarbamide and underwent leukapheresis. They were treated with nilotinib and imatinib respectively, resulting in haematological remission and improvement of their eye signs at 4 and 6 weeks, respectively (Figures 1C,D and 2C,D).

The fundus findings illustrate two pathophysiological responses to CML. The first case depicts decompensated leukaemic retinopathy. Hyperviscosity and endothelial dysfunction leads to vessel rupture, intra‐retinal haemorrhage and fibrin‐platelet plugging at the site of rupture (ie Roth Spots), as well as perivascular sheathing and cystoid macular oedema. The second case demonstrates a hyperdynamic retinal circulation and optic disc swelling, which are likely to be an adaptive (and fully compensated) local circulatory response to the patient's chronic anaemia.

The detection of Roth spots or optic disc swelling should lead to systemic work‐up of the patient. Clinicians are alerted to the fact that these findings may be indicative of leukaemic disease requiring prompt, potentially lifesaving, treatment.

## CONFLICT OF INTEREST

The authors declare that there is no conflict of interest that could be perceived as prejudicing the impartiality of the research reported.

## ETHICS STATEMENT

The report adhered to the ethical principles outlined in the Declaration of Helsinki as amended in 2013.
